# Defective Black TiO_2_: Effects of Annealing Atmospheres and Urea Addition on the Properties and Photocatalytic Activities

**DOI:** 10.3390/nano11102648

**Published:** 2021-10-09

**Authors:** Xu Zhang, Min Cai, Naxin Cui, Guifa Chen, Guoyan Zou, Li Zhou

**Affiliations:** 1 Institute of Eco-Environmental and Plant Protection, Shanghai Academy of Agricultural Sciences, Shanghai 201403, China; zhangxu@saas.sh.cn (X.Z.); caimin@saas.sh.cn (M.C.); cuinaxin@saas.sh.cn (N.C.); 13321836112@163.com (G.C.); zouguoyan@263.net (G.Z.); 2 Shanghai Engineering Research Center of Low-Carbon Agriculture, Shanghai 201403, China

**Keywords:** black TiO_2_, annealing atmosphere, urea, oxygen vacancy, photocatalytic activity

## Abstract

A series of black TiO_2_ with and without the addition of urea were successfully prepared using a simple one-step synthetic method by calcination under different atmospheres (vacuum, He, or N_2_). The physicochemical, optical, and light-induced charge transfer properties of the as-prepared samples were characterized by various techniques. It was found that a vacuum atmosphere was more beneficial for the formation of oxygen vacancies (OVs) than the inert gases (He and N_2_) and the addition of urea-inhibited OVs formation. The samples annealed in the vacuum condition exhibited better visible-light adsorption abilities, narrower bandgaps, higher photo-induced charge separation efficiency, and lower recombination rates. Hydroxyl radicals (·OH) were the dominant oxidative species in the samples annealed under a vacuum. Finally, the samples annealed under vacuum conditions displayed higher photocatalytic activity for methylene blue (MB) degradation than the samples annealed under He or N_2_. Based on the above, this study provides new insights into the effects of annealing atmospheres and urea addition on the properties of black TiO_2_.

## 1. Introduction

Titanium dioxide (TiO_2_) is the most widely investigated photocatalyst for use in solar-driven environmental purification. However, conventional white TiO_2_ has poor visible-light absorption and rapid electron-hole recombination, resulting in unsatisfactory photocatalytic activity in solar-driven reactions [1]. Therefore, many efforts have been expended to harvest visible photons and improve the photocatalytic activities of TiO_2_-based materials. Various strategies have been employed to improve the optical and electronic properties of white TiO_2_, including the replacement of Ti^4+^ ions by metal ions, and the replacement of O^2−^ ions by non-metal ions in the TiO_2_ lattice, which has been able to extend the photoresponse of TiO_2_ to the visible-light region [2]. The chemical doping of TiO_2_ exhibited various colors because of the new band introduction by chemical dopants. However, the introduced dopants, acting as charge carrier recombination centers, affected the photocatalytic efficiency of doped TiO_2_ [3]. Consequently, appealing approaches based on dopant-free were proposed to overcome the above limitation. Chen et al. firstly reported that hydrogenation was used to produce black TiO_2_ to improve visible light absorption and photocatalytic activity without chemical doping [4]. In recent years, black TiO_2_ has triggered an explosion in interest for many environmental applications, due to its absorption of both visible and near-infrared solar light without the need for the addition of dopants to alter its properties.

The synthesis methods of black TiO_2_ include hydrogenation, chemical reduction, electrochemical reduction, laser ablation (in liquid), and oxidation approaches [5]. Most synthesis routes are based on the reduction of pristine TiO_2_. However, most reported reduction methods suffer from some inevitable drawbacks, including the requirements of high temperatures or pressures, toxic reagents, and complex operation processes with long time consumptions and high operational costs. Therefore, some studies have developed facile and low-cost strategies to synthesize black TiO_2_. Indeed, it is well known that heating TiO_2_ under vacuum conditions or a reducing atmosphere (i.e., H_2_, CO, and NH_3_) can lead to color changes [2,6]. The inert gas atmosphere has been considered as a reductant due to the formation of defective TiO_2-x_ under an Ar or N_2_ atmosphere. The disordered layers form only if crystallization is performed in an oxygen-free environment [7]. Some studies have revealed that black TiO_2_ prepared by sintering P25 under a vacuum showed comparable performance with black TiO_2_ prepared under hydrogen [8]. Huang et al. investigated TiO_2_ nanorod arrays annealed under different atmospheres (O_2_, air, N_2_, and Ar), and found that samples annealed under Ar with the highest oxygen vacancies (OVs) concentration exhibited the highest photocurrent density [9]. Understanding the significant roles of calcination atmospheres on properties of produced black TiO_2_ would hold the key to the mechanism of the superior performances.

Although these synthesized black TiO_2_ have demonstrated improved performance, the explanations are largely diverse. It was widely accepted that TiO_2_ with changed colors exhibited different structural (lattice modification or disorder), chemical (formation of Ti^3+^, OVs, etc.), and physical properties (such as optical features), leading to different photocatalytic activities [10,11,12,13]. In contrast to the case of doping TiO_2_ that exhibited various colors because of the introduction of new band levels due to doped elements, extensive studies have revealed that the production of crystal and surface defects such as OVs or Ti interstitial is responsible for narrowing the bandgap of black TiO_2_ based on being dopant-free [11,14]. Meanwhile, others have reported that the key factor of bandgap narrowing was a unique core-shell structure with an oxygen-deficient disordered shell and a crystalline core [15]. It is commonly believed that the light-harvesting ability of a semiconductor is intimately associated with its bandgap and its subsequent photocatalytic performance. However, in many cases, although a narrower bandgap is beneficial for light absorption, the photoactivity of black TiO_2_ does not match its attractive absorption spectra [2,16]. To date, despite various efforts to study black TiO_2_ under various synthesization conditions, controversy persists regarding the mechanism of narrowed band gaps and the improved photoactivity of black TiO_2_.

Here, we synthesized black TiO_2_ using facile calcination under a vacuum and inert atmosphere (He and N_2_). Urea was added as a foreign dopant into the Ti precursor before the calcination process. The synthesized black TiO_2_ was characterized in detail to analyze the chemical and physical properties, and the electronic band structures of the materials were calculated. The production of reactive oxygen species (ROS) in the photocatalytic processes was determined to verify band structures and mid-gap states. MB decomposition was used as a model reaction to examine the photocatalytic activities of all forms of black TiO_2_. The two questions that we aimed to answer in this study were: (1) How do vacuums and inert atmospheres (He and N_2_) affect the properties of black TiO_2_? (2) Does the addition of urea benefit negatively impact the production and photoactivity of black TiO_2_?

## 2. Materials and Methods

### 2.1. Materials and Reagents

Tetrabutyl titanate (≥98%), urea (≥99.5%), hydrochloric acid (36.0–38.0%), and absolute ethanol (≥99.7%) were purchased from Sinopharm Chemical Reagent Co. Ltd. (Shanghai, China). Commercial TiO_2_ (P25) powder was purchased from Degussa Corporation Germany. Deionized (DI) water was used throughout the experiment.

### 2.2. Photocatalyst Synthesis

The synthesis process has been reported in our previous work [17]. Briefly, tetrabutyl titanate (14 mL) and urea (0.6 g) were mixed in ethanol (20 mL) using a magnetic stir bar (500 rpm) to form a light-yellow transparent solution A. Then, solution B, a mixture of ethanol (10 mL), DI water (5 mL), and hydrochloric acid (1 mL), was added dropwise into solution A, and a glass rod was used to stir the mixture until a white colloid formed due to a hydrolysis procedure. The white colloid was dried at 80 °C overnight. The resulting material was ground carefully and the obtained white powder was calcined under a vacuum, He, or N_2_ at 500 °C for 2 h at a heating rate of 4 °C min^−1^. For comparison, the samples without urea addition were also calcined under different atmospheres at the same conditions. The obtained materials with urea addition calcined under vacuum conditions, He, and N_2_ were denoted as U-T-V, U-T-He, and U-T-N_2_, respectively. The materials without urea addition calcined under vacuum conditions, He, and N_2_ were denoted as T-V, T-He, and T-N_2_, respectively.

### 2.3. Material Characterization

The surface morphologies of samples were characterized by field-emission scanning electron microscopy (SEM, JEOL-JSM-6700, Tokyo, Japan). High-resolution transmission electron microscopy (HR-TEM) was performed with JEOL-JEM-2100 microscopy (Tokyo, Japan). The crystalline phases of the as-prepared samples were analyzed by powder X-ray diffraction (XRD, Bruker D8 Advance, Germany), with Cu-Ka radiation source (λ = 0.15406 nm) in the range of 10–90° at 40 kV and 40 mA. Surface electronic states of the samples were measured by X-ray photoelectron spectroscopy (XPS) using an upgraded RBD PHI-5000C ESCA system (PerkinElmer) with Mg Kα radiation (*h* = 1253.6 eV). Raman spectra were obtained by a Raman Microscope (HORIBA LabRam HR Evolution) equipped with a 514 nm laser excitation source. UV-visible diffuse reflectance spectra (DRS) of the samples were acquired by a LAMBDA 950 spectrometer (PerkinElmer), with an integrating sphere and BaSO_4_ as a reference. Electron paramagnetic resonance (EPR) measurements of surface species of as-prepared powder samples were performed on a JEOL JES-FA200 spectrometer at room temperature, with a resonance frequency of 9.43 GHz and microwave power of 1.0 mW. EPR was also used to obtain signals of radical spin-trapped by spin-trap reagent 5,5-dimethyl-1-pyrroline N-oxide (DMPO). All EPR samples containing 10 mg photocatalyst in 5 mL DMPO solution (10 mM) were placed in a ~1 mm capillary, in which DMPO+water and DMPO+methanol systems were studied. The samples were irradiated by 300 W Xenon light (λ = 400–980 nm) for 1 min, and then the EPR spectra were obtained. The EPR instrumental settings were as follows: center field = 3365 Gauss, field sweep = 50 Gauss, sweep time = 1 min; microwave frequency, 9.4 GHz; microwave power, ~1.0 mW; modulation amplitude, 1.0 × 1000; time constant, 0.03 s; and receiver gain, 1 × 10^5^, 8192 points. Photoluminescence (PL) spectra were obtained with a fluorescence spectrophotometer (HORIBA MAX-4) using an excitation wavelength of 365 nm. The specific surface area and pore characteristics were determined by the N_2_ adsorption-desorption isotherm (ASAP2460, Micromeritics Instrument Corporation, USA) using the Brunauer–Emmett–Teller (BET) method and Barrett–Joyner–Halenda (BJH) method, respectively. Electrochemical impedance spectra (EIS) were measured by a three-electrode setup (CHI-760E, China). A Pt plate and Ag/AgCl were used as counter and reference electrodes, respectively, and 1 M KOH was used as the electrolyte. The working electrode was prepared by dropping 20 µL of catalyst suspension (10 mg of catalyst in 5 mL of glycerol) onto a 1.0 cm × 1.0 cm FTO glass. EIS was measured in the frequency range of 10–10^5^ Hz, with an AC voltage amplitude of 0.5 V.

### 2.4. Photocatalytic Activity for Degradation of Organic Pollutants

The photocatalytic activities of the TiO_2_ samples were evaluated by the degradation of MB under simulated solar light irradiation. The experiments were conducted in a multiposition photochemical reactor (CEL-LAB500E4, Beijing China Education Au-light Co., Ltd., Beijing, China). A 500-W xenon lamp was placed into a condensation tube in the center of six 100 mL-quartz tubes. The light intensities were kept at 45.3 mW cm^−2^ under simulated solar light irradiation. The condensed water was recycled constantly to keep the experimental system cool. The photocatalyst (0.04 g) was added into a 100 mL MB solution (10 mg L^−1^) and stirred for 0.5 h in the dark to reach adsorption–desorption equilibrium between the photocatalyst and the MB. After turning on the light, 5 mL of suspension were taken out at different intervals of time between 30–60 min. After centrifugation, the MB concentration was monitored by measuring the absorbance of the solution at 664 nm using a UV-vis spectrophotometer (UV-1801, BFRL, Beijing, China).

## 3. Results and Discussion

### Morphological and Structural Characteristics of the Photocatalysts

**Figure 1:**
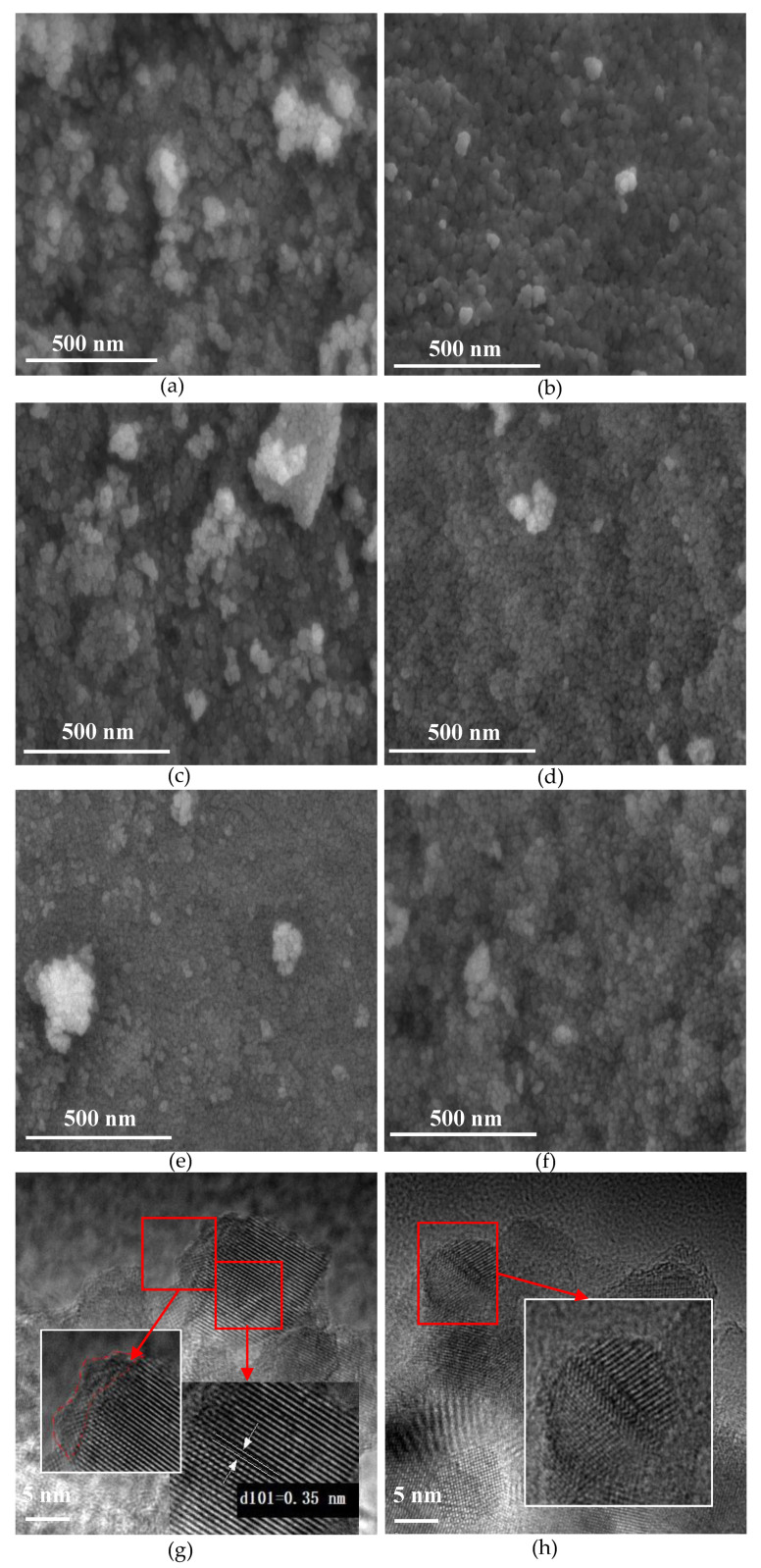
SEM of (**a**) T-V, (**b**) U-T-V, (**c**) T-He, (**d**) U-T-He, (**e**) T-N_2_, and (**f**) U-T-N_2_, as well as (**g**,**h**) HR-TEM of T-V.

The SEM images of as-prepared black TiO_2_ samples show an agglomeration of irregular primary nanoparticles, as shown in Figure 1a–f. For the samples annealed under a vacuum and He atmosphere, the nanoparticles without urea addition were fluffier than those with urea addition. However, the samples exhibited opposite characteristics when annealing under N_2_, and the sample without urea addition is flatter and more compact. The particle sizes in the SEM images were analyzed (Figure S1), and the relative data is shown in Table 1. The particle sizes of the as-prepared black TiO_2_ were 13–21 nm. The samples with urea addition exhibited larger particle sizes than those without urea addition for all atmospheres. The annealing atmosphere can also affect the particle size, and the N_2_ atmosphere leads to smaller particle sizes. U-T-V had the largest particle size and T-N_2_ had the smallest particle size. As shown in Figure S2, the lattice fringe of approximately 0.35 nm corresponding to the (101) plane of anatase TiO_2_ can be observed in all as-prepared black TiO_2_ samples, which indicates that anatase TiO_2_ is the majority of materials [18]. As shown in Figure 1g,f, the disordered surface and lattice deformation were owed to the unsaturated Ti atoms derived from the OVs [4,19]. In the TEM images of other TiO_2_ samples, disordered surface and lattice deformation are also observed, indicating defect formation in all as-prepared black TiO_2_ samples (Figures are not shown here). This proves that defects can be formed when annealed under a vacuum or inert atmosphere. The existence of defects like OVs can be confirmed by follow-up analyses.

As shown in Table 1 for the samples annealed under He or N_2_, a smaller material grain size provided a higher specific surface area and a smaller adsorption average pore diameter. However, for the samples annealed under vacuum, the sample with urea addition showed a higher specific surface area, which was possibly ascribed to the higher carbon content (3.5%) when compared with other samples (less than 3.0%) (the data from SEM-EDS in Table S1). The texture characteristics, such as the specific surface area and particle size, may affect the photocatalytic activity of the materials.

**Figure 2:**
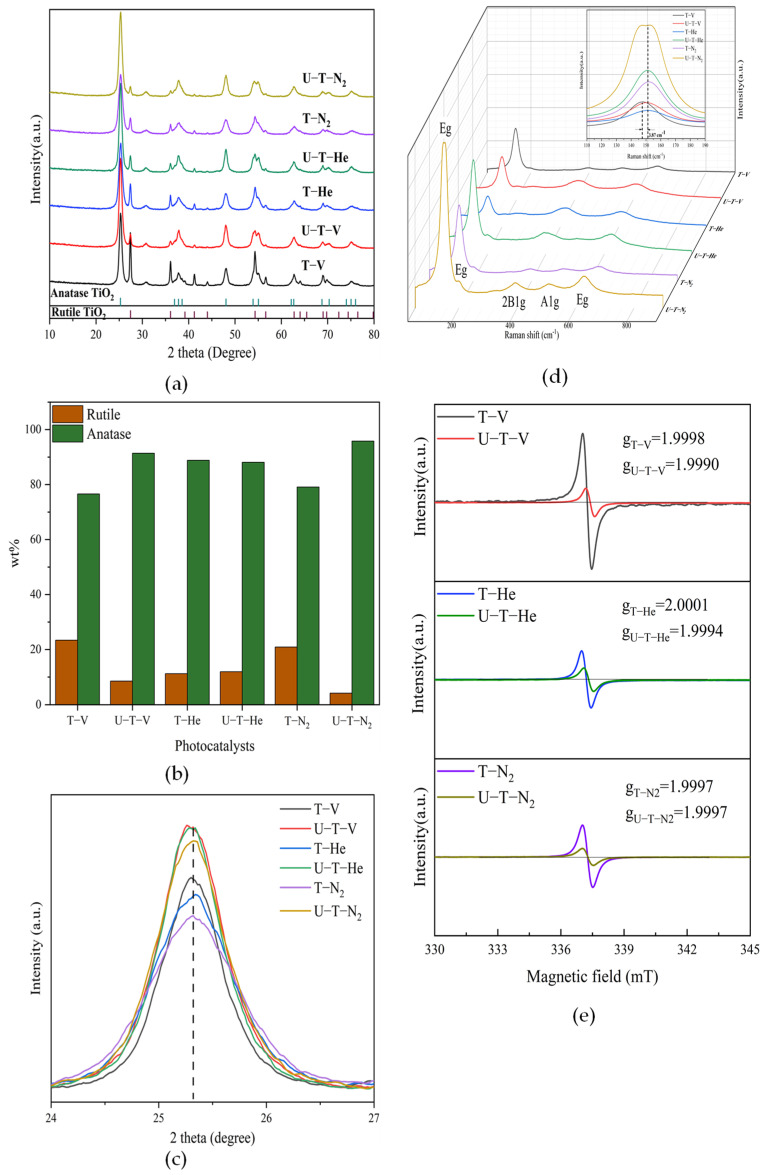
(**a**) XRD patterns of the as-prepared TiO_2_ samples; (**b**) The weight contents (wt%) of rutile and anatase TiO_2_ in different samples obtained from the integrated intensities of the anatase (101) and rutile (110) diffraction peaks by a reference intensity ratio (RIR) method [20]; (**c**) The most intense peak (101) of XRD patterns associated with different samples; (**d**) Raman spectra of different samples and the inset is the most intense peak (E_g_ (1)) of Raman spectra associated with different samples; (**e**) EPR spectra of different materials.

The XRD patterns of the as-prepared black TiO_2_ samples are presented in Figure 2a. The diffraction peaks of all samples were consistent with the standard pattern of anatase TiO_2_ (JCPDS No. 21-1272) and rutile TiO_2_ (JCPDS No. 21-1276), indicative of the presence of anatase and rutile TiO_2_ for all samples. The quantitative phase analysis was calculated from the integrated intensities of (101) peak for anatase phase and (110) peak for rutile phase using a reference intensity ratio (RIR) method [20]. As shown in Figure 2b, the weight percentage of the anatase phase was much higher than that of the rutile phase, which indicates anatase TiO_2_ was the majority of as-prepared samples. The U-T-N_2_ sample contains the least rutile TiO_2_ phase (4.2%), and the T-V sample contained the most rutile TiO_2_ phase (23.4%). Except for the samples annealed under He atmosphere, the urea addition inhibited the transfer from anatase to rutile phase for samples annealed under vacuum or N_2_ atmosphere. The same results had been observed in our previous study [17]. From Figure 2c, the intensities and the full width at half maxima of (101) peaks in the samples with urea addition were sharper than those in the samples without urea addition, which indicated that crystal sizes became larger [21]. The crystal sizes of as-prepared samples are determined by the Scherrer formula (as shown in Table 1). The crystal sizes of the samples with urea addition were larger than those without urea addition, which was identical to the results from the analysis of the (101) peak. From Figure 2c, the larger peak width of the samples without urea addition can also be derived from OVs and the resulting disorder-induced lattice strain, which indicated more OVs in the samples without urea addition than those with urea addition.

The phase of TiO_2_ can also be identified by Raman (as shown in Figure 2d,e). Six Raman-active peaks (3E_g_+2B1_g_+A1_g_) of anatase TiO_2_ were detected, which is consistent with the results of XRD. Compared with the Raman spectra of pristine anatase TiO_2_ located at 143, 196, 326, 395, 512, 645 cm^−1^ [22], the Raman spectra of all as-prepared black TiO_2_ in this study occurred redshift, which could be ascribed to the lattice distortion and low crystallinity [23]. In other words, the formation of OVs in all samples was confirmed by Raman spectra. As shown in Figure 2e, T-He and T-N_2_ showed higher wavenumbers than U-T-He and U-T-N_2_, which was evidence of more OVs in the samples without urea addition than in the samples with urea addition and annealed under He and N_2_ [23]. The results are consistent with the EPR signals in Figure 2f. It was noted that the Raman shifts of the samples annealed under a vacuum did not support the amount of formed OVs. The Raman spectra of U-T-V occurred red shift compared with that of T-V. However, the sample T-V showed a higher EPR signal area. On the other hand, the intensities of Raman signals showed different trends for samples annealed under vacuum or inert atmospheres. For the samples with urea addition annealed under inert atmospheres, the Raman intensities are higher than those without urea addition, which confirms that there are more OVs without urea addition than with urea addition, due to the inverse relationship between the Raman intensity and the amount of OVs [24]. For the samples with and without urea addition annealed under the vacuum condition, the Raman intensity is similar. This is evidence of the different effects of vacuum and inert atmospheres on the properties of black TiO_2_. The reasons for the differences will be explained in the following analysis.

The OVs states in the as-prepared black TiO_2_ were investigated by EPR, which identify unpaired electrons in paramagnetic species. The g-factor extracted from the EPR spectra provides a qualitative analysis of defect species. The g-factors were calculated using the formula: g = 71.44ν/Β, where ν is the microwave frequency of X—band spectrometer (GHz) and B is the applied magnetic field (mT). All calculated g-factors are displayed in Figure 2d. As can be seen in Figure 2d, the obvious symmetrical signals at g≈2.000 were detected in all samples, which can be ascribed to the formation of defects in samples [25]. The g-factors of the samples with urea addition annealed in a vacuum and He atmosphere experienced a slight shift compared with that without urea addition. In general, the deviation of g-value from a standard value for free-electron (g_e_ = 2.0023) and shape (symmetric or asymmetric) of the EPR signal provides direct evidence of intrinsic defective domains within the TiO_2_ lattice [26]. The g-values of <2.0023 with isotropic components (symmetric signal) in this study arise from OVs with one trapped electron [27]. On the other hand, the intensity of the signals reflects the relative concentration of the paramagnetic centers [28,29]. As shown in Figure 2d, the sample T-V exhibited the strongest signal. Concerning the three groups of samples annealed in different atmospheres, the sample without urea addition showed a higher EPR signal than that with urea addition. The results show that vacuum condition was more beneficial for OVs formation than the inert gases (He and N_2_) and that urea addition inhibited OVs formation.

On one hand, why was the vacuum condition more beneficial for OVs formation than the inert gas? It has been reported that OVs can be created by annealing the semiconductor oxides in an oxygen-deficient atmosphere, such as pure He, N_2_, or vacuum conditions [28]. The formation of OVs in the TiO_2_ samples under vacuum or oxygen-deficient atmosphere can be ascribed to the removal of the oxygen in the lattice (O_L_) of TiO_2_, which can be demonstrated by an equilibrium reaction (Equation (1)) [30]:

O_L_ = 1/2O_2_(g) + OV + 2e^−^ (1)

It can be deduced that the concentration of OVs increases with a decrease in O_2_ pressure. The oxygen-depleted condition under thermal annealing would facilitate the formation of OVs [31]. The vacuum condition is, therefore, better than inert gas for oxygen gas release due to its lower pressure, which causes the formation of the most OVs in the sample T-V.

On the other hand, why did the addition of urea inhibit the formation of OVs? Our previous study revealed that the addition of urea addition as an N precursor inhibited the incorporation of N and the production of OVs [17]. Nevertheless, it had been previously revealed that N doping facilitates the formation of OVs [32]. In other words, the urea addition inhibited the N doping directly and then decreased the production of OVs. Furthermore, some researchers have reported that it was more difficult for N atoms to enter the TiO_2_ lattice for the doped TiO_2_ samples prepared from a one-step reaction of crystalline TiO_2_ and N-containing precursor [2], just like in this study. Therefore, it needs to be made clear whether the N was incorporated into the lattice of black TiO_2_ successfully for the samples with urea addition, which can be concluded from the following XPS study.

**Figure 3:**
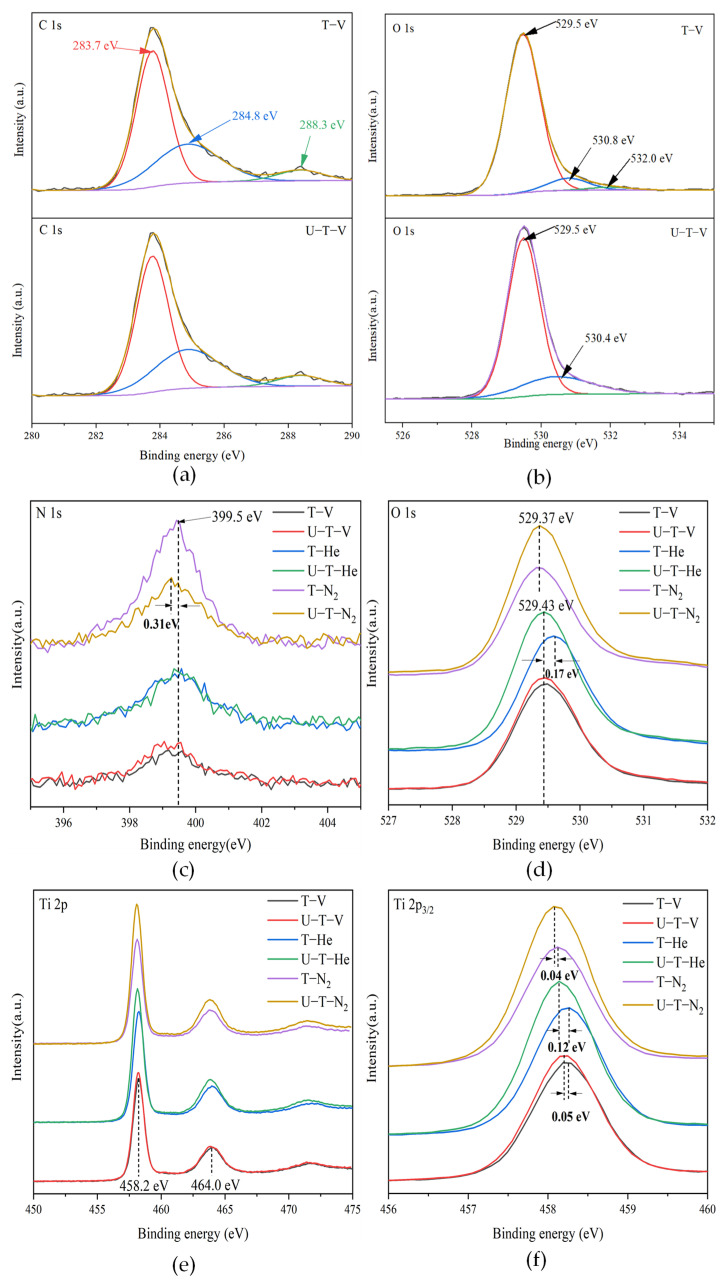
High-resolution XPS of (**a**) C 1s of T-V and U-T-V; (**b**) O 1s of T-V and U-T-V; (**c**) N 1s; (**d**) O 1s; (**e**) Ti 2p; (**f**) enlarged Ti 2p3/2 of the as-prepared samples.

XPS was performed to analyze the chemical states of as-prepared samples. The C–C peak at 284.8 eV was used as a charge correction reference. Taking the samples of T-V and U-T-V as examples, there were common peaks located at 284.8 eV for the C 1s spectra of the samples T-V and U-T-V, as shown in Figure 3a, which can be attributed to surface carbon with C–C bond due to carbon from the internal standard used during the XPS measurements. Some studies suggested that the peak at 288.3 eV was assigned to the C–N bond from the nitrogen precursor, like urea in this study [33]. It was noticed that the peak at 288.3 eV exists in the C 1s spectra of both T-V and U-T-V samples, while there was no nitrogen source added to the T-V sample. Therefore, it is possible that the C–N bond came from the N impurity in the measurement environments, rather than the added urea itself. The urea addition did not affect the production of C–N, which can be analyzed again by the XPS spectra of N 1s. No peak at ca. 281 eV associated with the Ti–C bond was detected, suggesting that oxygen was not substituted by carbon in the lattice of black TiO_2_ [34]. As shown in Figure 3c, the samples, except U-T-N_2_, display only one N 1s core level peak at around 399.5 eV, indicating that most samples contain uniform N species. In some studies, the main peak of N 1s, located at 399.5–399.7 eV, contributed to the substitutional N in N–Ti–O [35,36], where the N atoms replaced some lattice oxygen. Some other studies revealed that the peak at about 400 eV could be assigned to molecularly chemisorbed γ-N_2_ [37]. In this study, the samples without urea addition do not contain an N source during synthesis processes, thus the peak of N 1s at about 399.5 eV could be attributed to the chemisorbed N_2_ in the environment. The intensities of N 1s for the samples annealed in the N_2_ atmosphere are higher than that annealed in a vacuum and He atmosphere, which confirms the N_2_ absorbed on the surface of materials. There is a slight blue shift of 0.31 eV for the N 1s in the U-T-N_2_ sample, indicating the formation of other N-containing substances. In some studies, the peak of N 1s located at 399.2 eV can be ascribed to amines (NH*_x_*) [38], which is from the decomposition of urea under the N_2_ atmosphere. According to the XPS spectra of O 1s (as shown in Figure 3c), all the samples show lower-energy shifts than the O 1s located at 529.9 eV in the Ti–O linkages of TiO_2_. The conclusion of OVs formation can be obtained from the lower-energy shift of O 1s peaks compared with that of the pristine TiO_2_ [39]. For the samples annealed under vacuum, both O 1s spectra showed two peaks, as shown in Figure 3b. The major peak located at around 529.5 eV could be associated with the lattice oxygen in TiO_2_, which is the characteristic of the chemical bonding between metal and oxygen atoms. The other peak located at around 531.0 eV corresponded to the surface absorbed oxygen, which has a variety of origins, such as O^−^, O_2_^−^ and O^−2^ [40]. These adsorbed oxygen species in XPS spectra are derived from the formation of OVs in TiO_2_. Thus, the relative density of OVs can be quantitatively estimated by measuring the relative intensity ratio (RIR) of O 1s (lattice oxygen)/O 1s (adsorbed oxygen) [25,41]. The estimated RIRs of T-V and U-T-V are 3.43 and 3.54, respectively. The increase of RIR for U-T-V indicates fewer OVs than that in T-V, which is consistent with the EPR results.

Figure 3e shows the characteristic Ti 2p peaks of all samples. The Ti 2p peaks of T-V located at 464.0 eV and 458.2 eV, correspond to Ti 2p1/2 and Ti 2p3/2, respectively. Compared with the Ti 2p peaks (464. 6 and 458.8 eV for Ti 2p1/2 and Ti 2p 3/2) of pristine TiO_2_, the peaks of Ti 2p in all black TiO_2_ shifted to lower energy. A shift of Ti 2p to lower binding energy is owed to the OVs formation, which is consistent with previous studies [39,42]. Concerning enlarged Ti 2p3/2 peaks of all samples in Figure 3f, the samples with urea addition (U-T-V, U-T-He, and U-T-N_2_) show lower-energy shifts of 0.05, 0.12, and 0.04 eV, respectively, compared with the samples without urea addition (T-V, T-He, and T-N_2_), indicating an increase in electron density around Ti atoms after urea addition. In conclusion, the XPS results reveal the formation of OVs and no N or C incorporation in the lattice of black TiO_2_.

**Figure 4:**
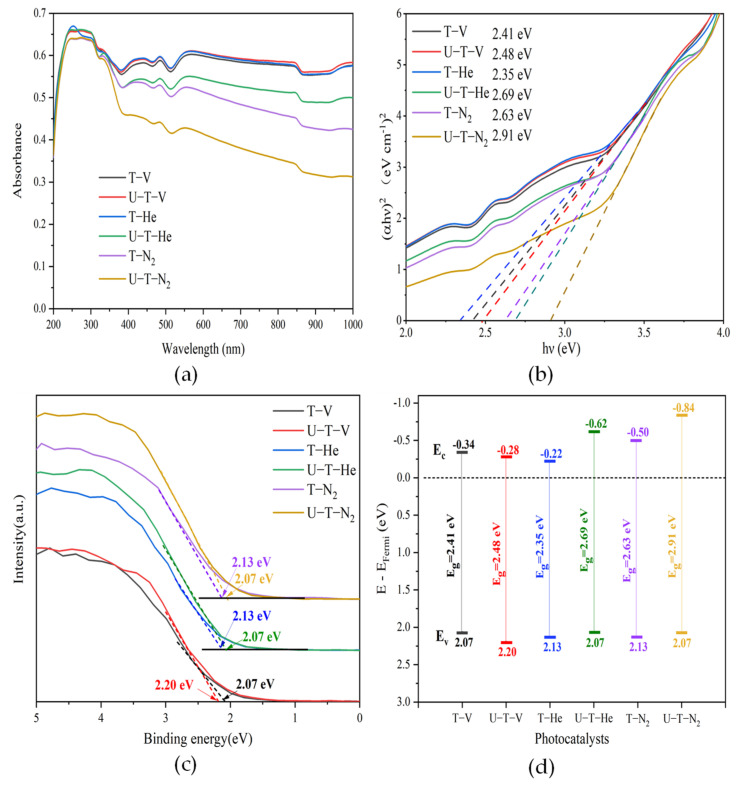
(**a**) UV-vis spectral absorbance; (**b**) bandgap energies evaluated from the plots of (*αhν*)^2^ versus *hν*; (**c**) XPS valence band (VB) spectra; (**d**) the calculated band energy.

Figure 4a shows the UV-vis spectral absorbance of the samples. The TiO_2_ samples annealed under inert gas or vacuum conditions exhibited a broad light absorption in the range of 200–1000 nm. The samples of T-V, U-T-V, and T-He displayed much stronger light absorption than the other three samples. The strong light absorption of the samples can be attributed to the disordered surface layer due to the OVs formation [43], which was observed from the TEM images. The bandgaps can be evaluated from the plots of (*αhν*)^2^ versus *hν*, as shown in Figure 4b. The bandgap energies were estimated to be 2.41, 2.48, 2.35, 2.69, 2.63, and 2.91 eV for T-V, U-T-V, T-He, U-T-He, T-N_2_, and U-T-N_2_, respectively. The formation of OVs by the O^2−^ replacement in the regular lattice of TiO_2_ induced a donor level below the conduction band (CB), which is normally at 0.75–1.18 below the CB of TiO_2_ [31], resulting in a narrower bandgap of the samples. Figure 4c exhibited the XPS spectra of VB of the samples, where the energies of VB (E_v_) (the difference of the valence band edge below the Fermi energy) are 2.07, 2.20, 2.13, 2.07, 2.13, and 2.07 eV for T-V, U-T-V, T-He, U-T-He, T-N_2_, and U-T-N_2_, respectively. It should be noted that the urea addition did not influence the VB energy. The materials annealing under N_2_ exhibited a higher position of VB than those annealed under vacuum or He atmosphere. As is well known, OV does not influence the VB [42]. On the other hand, according to the XPS results of N 1s, there was no N doping in the samples. How did the shift of VB states happen? As is observed in the TEM images, the disordered layer and lattice deformation existed in all samples. On-lattice disorders in TiO_2_ nanocrystal induced midgap states which upshifted the VB edge of TiO_2_ nanocrystals, accompanied by a reduced bandgap [4]. For the samples annealed under He and N_2_, the introduced urea benefited the disordered layer and lattice deformation, and then upshifted the VB edge higher. However, for the samples annealed under the vacuum condition, the sample without urea addition exhibited a higher upshift. Based on the energies of the bandgap and the VB of as-prepared samples, the electronic band structures of the samples are summarized in Figure 4d.

**Figure 5:**
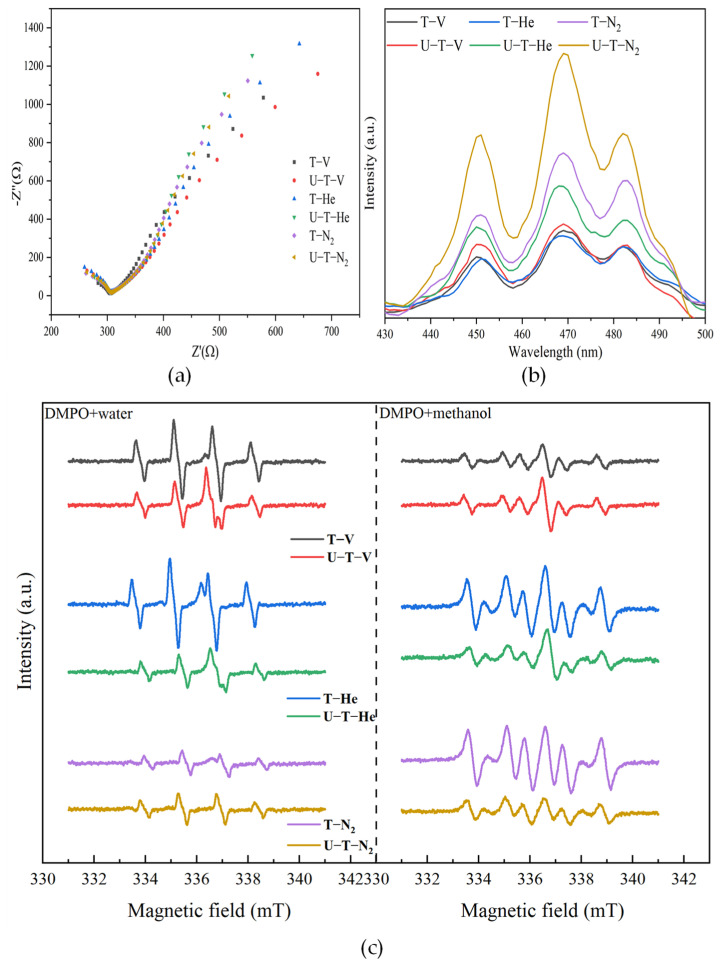
(**a**) EIS Nyquist plots of the samples; (**b**) PL emission spectra of the samples; (**c**) EPR spectra of DMPO adducts obtained in DMPO+water and DMPO+methanol system over the samples after visible-light irradiation for 1 min.

It should be noticed that ROS production depends not only on the band structure, but also on the separation and recombination efficiency of photo-induced electrons and holes under light irradiation. Therefore, based on the above characteristics of light absorption, bandgap, and band construction of all the samples, the separation and recombination efficiencies of light-induced charges are observed from the results of the EIS Nyquist plots and PL emission spectra, as shown in Figure 5a,b.

Figure 5a shows EIS Nyquist plots for all of the samples. The relative sizes of circular arc radii correspond to the charge transfer resistance and separation efficiency of photo-induced electrons and holes [44]. The relative arc sizes are in the following order: U-T-V > T-V > T-He. The other three samples exhibited similar but smaller arc sizes. The sample U-T-V showed the smallest circular arc radius, indicating that it has the highest electron-hole pair separation and electron transfer efficiency to electron donor/acceptor. Figure 5b shows the PL emission spectra of all the samples in the range of 430–500 nm with an excitation wavelength of 365 nm, resulting from the recombination of free carriers. The excitonic PL resulted from surface OVs and defects of the samples. All the emission spectra shapes are similar, and three main emission peaks are located at 451, 469, and 482 nm, respectively. The peaks at 451 and 469 nm are attributed to band edge free excitons, and the peak at 482 nm is attributed to bound excitons [45]. The intensity of the peak at 469 nm reached the summit of the survey spectrum, demonstrating the formation of OVs in the lattice of the samples [46]. The samples annealing under the N_2_ atmosphere showed the highest emission intensity. T-V, U-T-V, and T-He exhibited similar but lower emission intensities, indicating a reduced recombination rate of photogenerated electrons and holes in the above three samples. The results agreed well with the results of the EIS test shown in Figure 5a. T-V, U-T-V, and T-He displayed faster interfacial charge transfers and lower charge recombination rates, which are the basic benefits of higher photocatalytic activities.

Except for the charge separation and transfer, the formation of ROS is also attributed to band structure. Based on the band structures of all samples shown in Figure 4d, the production of ·OH and ·O_2_^−^ has been proposed in the above analysis. As shown in Figure 5 in the DMPO+water system, the EPR spectra measured after irradiation of the samples in water with DMPO exhibited a quartet with hyperfine coupling constants, A_N_ = A_H_ = 1.50 mT. The values are consistent with reported values for the DMPO-·OH spin adduct [47]. In the DMPO+methanol system, the EPR spectra measured after irradiation of the samples in methanol with DMPO were different from DMPO-·OH. Evaluated hyperfine coupling constants A_N_ = 1.53 mT, A_H_ = 2.16 mT suggest the generation of the superoxide radical adduct DMPO-·CH_3_ [48], which is a strong indicator of ·OH, since ·OH could react rapidly with DMPO to yield DMPO-·CH_3_ at the expense of DMPO-·OH [48]. Here the EPR results of DMPO-·OH DMPO-··CH_3_ confirmed the production of ·OH. According to the intensity of the DMPO-·OH and DMPO-·CH_3_ signals, the sample T-V, U-T-V, and T-He generated more ·OH than the other samples. The photocatalytic activities of as-prepared samples for oxide organic pollutants depend on the production of ROS.

**Figure 6:**
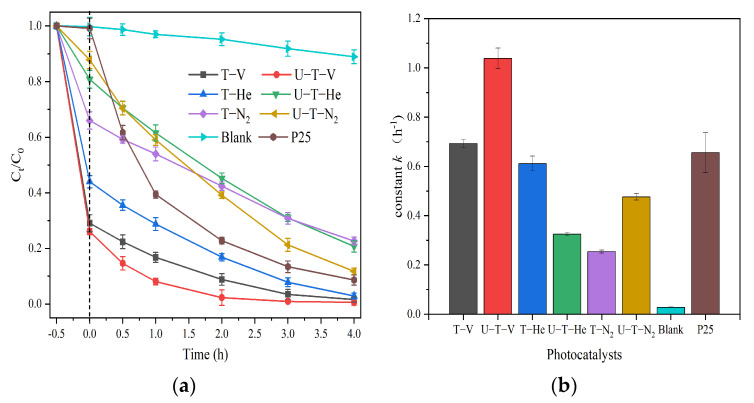
(**a**) Photocatalytic degradation activity for MB by the as-prepared TiO_2_ samples under simulated solar light irradiation; (**b**) Constant k of the fitted Pseudo-first-order dynamic curves of photocatalytic degradation for MB.

The photocatalytic activity of the samples was determined by the degradation of MB under simulated solar light irradiation. Before conducting a photocatalytic activity investigation, it is essential to study the adsorption ability for MB onto the surface of the samples, due to its pivotal role in further photocatalytic degradation. All the samples exhibited different adsorption abilities for MB, with the following order: U-T-V > T-V > T-He > T-N_2_ > U-T-He > U-T-N_2_. The sample U-T-V displayed the best MB adsorption ability, with 69.36% of MB molecules adsorbed on the surface after 0.5 h of mixing in the dark, as shown in Figure S3a. After the simulated solar light irradiation, the MB concentrations in the solutions decreased rapidly, as shown in Figure 6a. The MB removal rates over the three samples T-V, U-T-V, and T-He were 98.38%, 99.40%, and 97.10%, respectively, after 4 h of irradiation under simulated solar light, which exhibited better MB degradation than pure P25, with a 91.34% removal rate. For the other three samples, i.e., U-T-He, T-N_2_, and U-T-N_2_, the MB removal rates were lower, at 79.25%, 77.34%, and 88.25%, respectively. The performance was further quantified with kinetic rate plots (as shown in Figure S3b) as a pseudo-first-order reaction. The calculated kinetic rate constants for the samples T-V, U-T-V, T-He, U-T-He, T-N_2_, U-T-N_2_, and P25 are 0.693 ± 0.023, 1.038 ± 0.047, 0,612 ± 0.036, 0.325 ± 0.009, 0.255 ± 0.008, 0.477 ± 0.016, and 0.656± 0.032 h^−1^, respectively, with an order of U-T-V > T-V > P25 > T-He > U-T-N_2_ > U-T-He > T-N_2_. The U-T-V sample exhibited the highest kinetic rate, which was 3.9 times higher than that of T-N_2_, which had the lowest kinetic rate.

The samples annealed under a vacuum atmosphere displayed the best photocatalytic activities for MB degradation compared with the samples annealed under He or N_2_ atmospheres, as a result of better MB adsorption capacities and visible light absorption abilities, production of more OVs, narrower bandgaps, a more positive VB position, more efficient charge separation, and transfer, and production of more ·OH. For the samples that annealed under the vacuum condition, the urea addition enhanced the MB adsorption capacity and degradation efficiency, which is potentially due to the larger surface area and higher efficiency of charge separation and transfer than that without urea addition.

## 4. Conclusions

A series of black TiO_2_ with or without urea addition were synthesized by calcination under different atmospheres (vacuum, He, or N_2_). The vacuum condition was more beneficial for the formation of OVs than the inert gases (He and N_2_) and urea addition inhibited OVs formation. There are no elements (N and C) incorporated into the lattice of black TiO_2_. It is the produced OVs and disordered layers that narrow the bandgap of black TiO_2_ via downshift of CB and upshift of VB. The samples annealed under the vacuum condition exhibited better visible-light adsorption abilities, narrower bandgaps, higher photo-induced charges separation efficiency, and lower recombination rates. The ·OH was the dominant oxidative species for the samples, and the samples annealed under the vacuum condition produced more ·OH than the other samples annealed under N_2_ atmosphere. Based on the differences between the as-prepared samples, the materials annealed under vacuum displayed higher photocatalytic activity for MB degradation compared with other materials. For the series of black TiO_2_, the material with urea addition annealed under vacuum exhibited the highest kinetic rate, which was 3.9 times higher than that of the material without urea addition under N_2_ owning the lowest kinetic rate. In general, the vacuum condition was better than inert atmospheres (He and N_2_) at synthesizing black TiO_2_, and urea addition enhanced photocatalytic activity, although it reduced the production of OVs.

## Figures and Tables

**Table 1:** Characteristics of the as-prepared TiO_2_ samples.

Samples	Particle Size ^1^ (nm)	Crystal Size ^2^ (nm)	BET Surface Area (m^2^/g)	Adsorption Average Pore Diameter (nm)
T-V	15 ± 3	11.1	55	7.0
U-T-V	21 ± 3	11.2	68	7.1
T-He	13 ± 3	8.8	71	6.2
U-T-He	17 ± 2	11.2	69	9.3
T-N_2_	13 ± 2	7.6	87	5.8
U-T-N_2_	16 ± 2	10.1	77	8.6

^1^ Measured from SEM; ^2^ Calculated from XRD with Scherrer Equation.

## Supplementary Materials

The following are available online at https://www.mdpi.com/article/10.3390/nano11102648/s1, Figure S1: Particle size distribution of (a) T-V, (b) U-T-V, (c) T-He, (d) U-T-He, (e) T-N_2_, and (f) U-T-N_2_., Figure S2: HR-TEM images showing lattice fringes of (a) T-V, (b) U-T-V, (c) T-He, (d) U-T-He, (e) T-N_2_, and (f) U-T-N_2_, Figure S3: (a) The adsorption capacity of as-prepared samples towards MB pollutant; (b) The fitting curves of ln(C_0_/C_t_) versus Time (h). Table S1: Element contents (wt%) of the as-prepared black TiO_2_ samples by SEM-EDS.
