# Supplementary material for: Defective Black TiO2: Effects of Annealing Atmospheres and Urea Addition on the Properties and Photocatalytic Activities
Source: Nanomaterials (Basel). 2021 Oct 9;11(10):2648. doi: 10.3390/nano11102648 (PMC8541354; doi:10.3390/nano11102648)
Supplement: Supplementary file 1 [file nanomaterials-11-02648-s001.zip › nanomaterials-1382116-supplementary.pdf]

nanomaterials

MDPI

Defective black TiO_2: effects of annealing atmospheres and urea addition on the properties and photocatalytic activities

Xu Zhang[1,2], Min Cai[1,2], Naxin Cui[1,2], Guifa Chen[1,2], Guoyan Zou[1,2], Li Zhou[1,2,*]

1 Institute of Eco-environmental and Plant Protection, Shanghai Academy of Agricultural Sciences, Shanghai, 201403, China; zouguoyan@263.net
2 Shanghai Engineering Research Center of Low-carbon Agriculture, Shanghai, 201403, China; zouguoyan@263.net
* Correspondence: joly.zhouli@gmail.com; Tel.: +86-18817365817 (L.Z.)

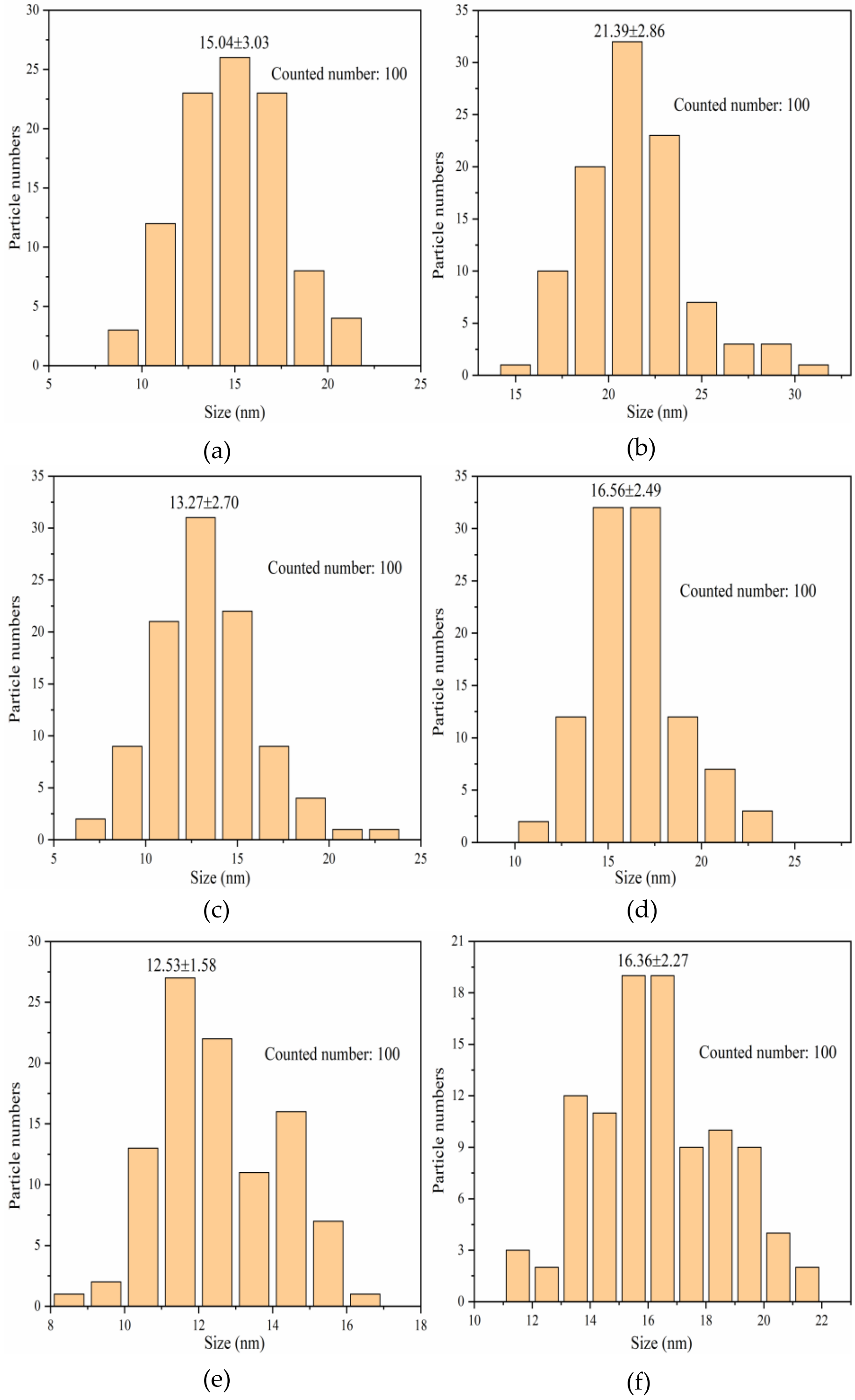

Figure S1. Particle size distribution of (a) T-V; (b) U-T-V; (c) T-He; (d) U-T-He; (e) T-N_2; and (f) U-T-N_2.

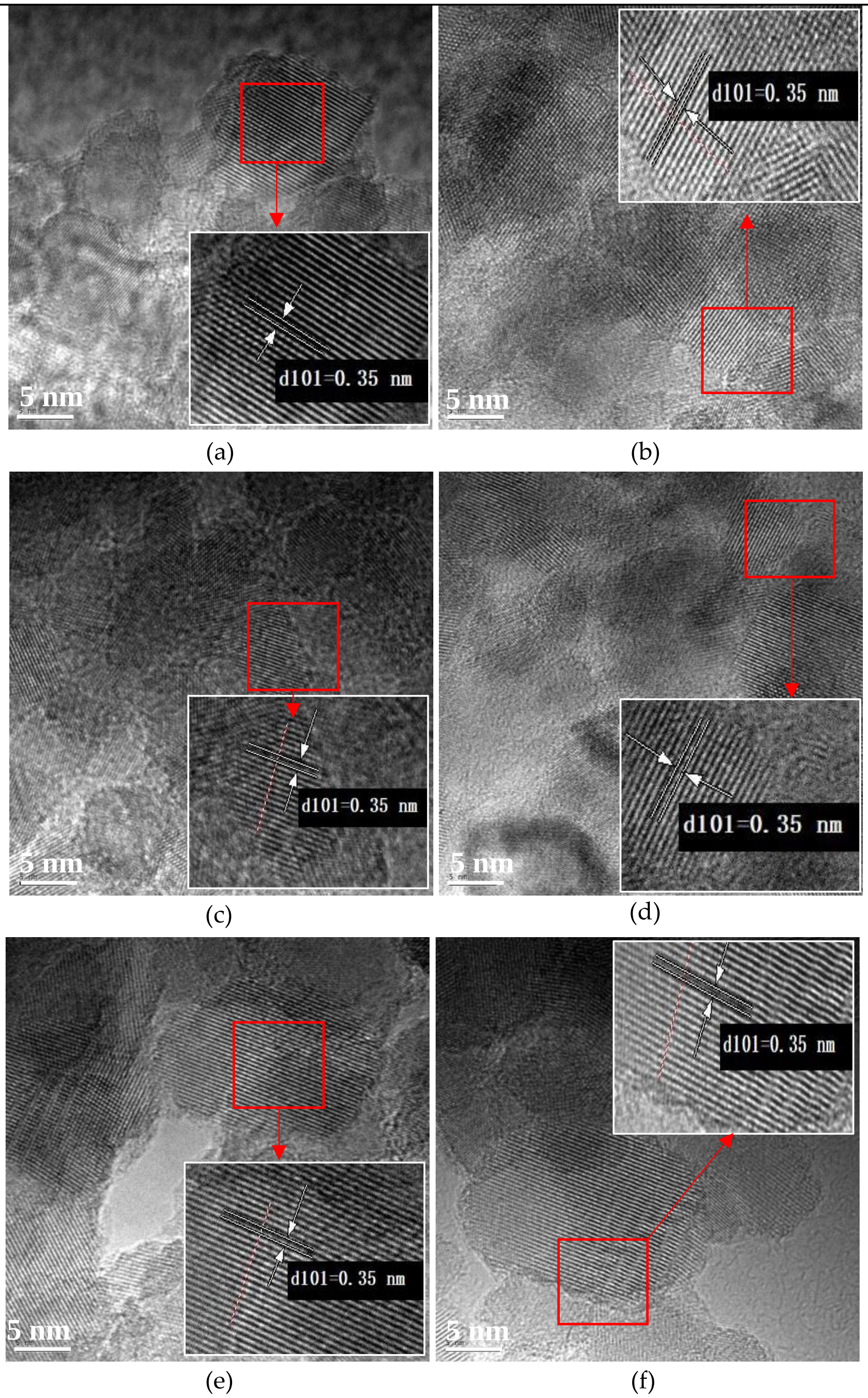

Figure S2. HR-TEM images showing lattice fringes of (a) T-V; (b) U-T-V; (c) T-He; (d) U-T-He; (e) T-N_2; and (f) U-T-N_2.

Table S1. Element contents (wt%) of the as-prepared TiO_2 samples by SEM-EDS.

Samples / Elements	T-V	U-T-V	T-He	U-T-He	$T-N_2$	$U-T-N_2$
C	2.02	3.53	2.91	2.45	2.48	1.75
N	0.91	0.86	1.40	1.26	0.96	1.79
O	21.62	26.83	28.56	24.99	23.50	27.21
Ti	75.45	68.77	67.13	71.30	73.06	69.25

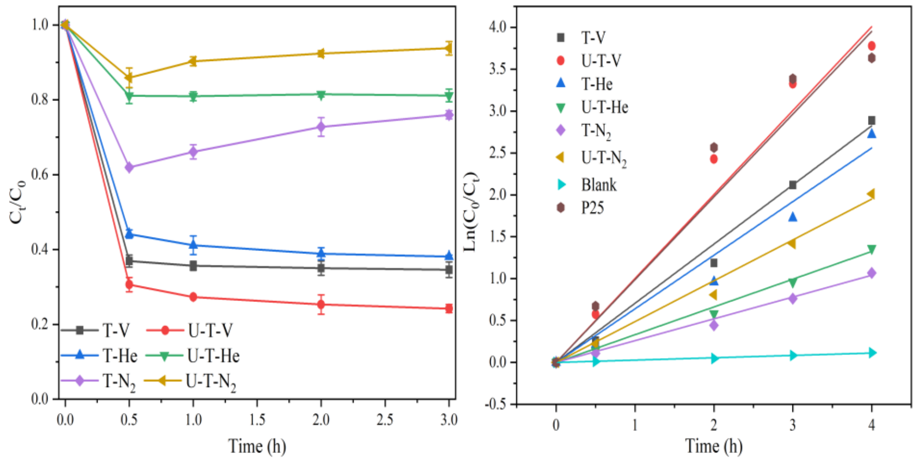

(a) (b)

Figure S3. (a) The adsorption capacity of as-prepared samples towards MB pollutant; (b) The fitting curves of $\ln(C_0/C_t)$ versus Time (h).
